# Morphology, Rheological and Mechanical Properties of Isotropic and Anisotropic PP/rPET/GnP Nanocomposite Samples

**DOI:** 10.3390/nano11113058

**Published:** 2021-11-13

**Authors:** Francesco Paolo La Mantia, Vincenzo Titone, Alessandro Milazzo, Manuela Ceraulo, Luigi Botta

**Affiliations:** 1 Department of Engineering, University of Palermo, Viale delle Scienze, 90128 Palermo, Italy; alessandro.milazzo02@unipa.it (A.M.); luigi.botta@unipa.it (L.B.); 2 INSTM, Consortium for Materials Science and Technology, Via Giusti 9, 50125 Florence, Italy; vincenzo.titone@unipa.it (V.T.); manuela.ceraulo@unipa.it (M.C.); 3 Irritec S.p.A., Via Industriale sn, 98070 Rocca di Caprileone, Italy

**Keywords:** nanocomposites, polymer blends, polypropylene (PP), graphene nanoplatelets (GnPs), polyethylene terephthalate (PET), rheology, mechanical properties

## Abstract

The effect of graphene nanoplatelets (GnPs) on the morphology, rheological, and mechanical properties of isotropic and anisotropic polypropylene (PP)/recycled polyethylene terephthalate (rPET)-based nanocomposite are reported. All the samples were prepared by melt mixing. PP/rPET and PP/rPET/GnP isotropic sheets were prepared by compression molding, whereas the anisotropic fibers were spun using a drawing module of a capillary viscometer. The results obtained showed that the viscosity of the blend is reduced by the presence of GnP due to the lubricating effect of the graphene platelets. However, the Cox–Merz rule is not respected. Compared to the PP/rPET blend, the GnP led to a slight increase in the elastic modulus. However, it causes a slight decrease in elongation at break. Morphological analysis revealed a poor adhesion between the PP and PET phases. Moreover, GnPs distribute around the droplets of the PET phase with a honey-like appearance. Finally, the effect of the orientation on both systems gives rise not only to fibers with higher modulus values, but also with high deformability and a fibrillar morphology of the dispersed PET phase. A fragile-ductile transition driven by the orientation was observed in both systems.

## 1. Introduction

Polymer blends are a convenient way to develop new materials, which combine the remarkable properties of different polymers [1,2], but several applications require the use of reinforced materials. For this reason, the incorporation of nanoparticles in a polymer matrix is often employed to enhance performance, and this has become an issue of interest in academia and industry [3,4]. In this respect, graphene has attracted a great deal of attention due to its capacity to improve the mechanical, thermal, and electrical properties of polymer nanocomposites and their behavior to thermomechanical and photooxidative degradation [5,6,7,8,9,10,11,12,13,14]. In addition, the presence of GnPs enhances the effect of elongated flow, which, consequently, improves mechanical properties with the draw ratio of nanocomposites compared to the matrix alone [9].

Polypropylene (PP) is a thermoplastic material widely used for many applications due to its low cost and processability. It is often blended with some high-melting-point polymers such as polyamides (PAs) [15,16,17] and polyethylene terephthalate (PET) [18,19,20,21], in order to improve high-temperature resistance of the blends, as well as being an economical and efficient way to improve the properties of PP [20]. In addition, in a few cases, different types of nano-fillers [22,23,24] have been used to increase the miscibility and minimize the phase separation of PP/PET blends.

In this work, the use of recycled PET derived from the common interest in recycling post-consumer plastics. There are only a few papers present in the literature on blends of PP and PET in presence of GnP.

Inuwa et al. [23] used styrene–ethylene–butylene–styrene-g-maleic anhydride (SEBS-g-MAH) and graphene nanoplatelets (GnPs), and reported their effect on the structure and mechanical properties of a PET/PP blend. The results of their study showed that the platelets remain intact and dispersed homogeneously in the polymer matrix without substantial exfoliation of GnPs. In addition, the improvements observed in mechanical properties were attributed to stiffness of the platelets and effective stress transfer between matrix and filler. The same authors, in another work [24], also studied the flammability characteristics of PP/PET/GnP nanocomposites. They reported that the improvement in flammability parameters can be attributed to the growth of aligned, dense, and organized char layers on the surface of the PP/PET/GnP nanocomposites. In addition, they found that the effective thermal conductivity of the nanocomposites increased with the increase in GnP loading.

The aim of this paper is to investigate the effect of GnPs on the morphology, rheological, and mechanical properties of a nanocomposite with a matrix of a blend of PP and rPET and, in particular, on the morphology and mechanical properties of melt-spun PP/rPET/GnP nanocomposite fibers with different draw ratios. The elongational flow strongly changes the morphology of the isotropic polymer system and, as a consequence, the final properties of the nanocomposite. To the best of our knowledge, the effect of the elongational flow and the orientation on this and other nanocomposite blends has not been reported in the literature.

## 2. Materials and Methods

The materials used in this work were a sample of polypropylene (PP; Hostalen PP H5416) in pellet form supplied by LyondellBasell (Houston, TX, USA) with melt flow index (MFI) of 0.3 g/10 min (2.16 kg at 230 °C), density of 0.897 g/cm^3^, and melting point of 139 °C, and a polyethylene terephthalate (PET) sample obtained from post-consumer water bottles. The MFI measured at 275 °C under a weight of 325 g (condition K) was 55 g/10 min [25].

Graphene nanoplatelets (GnPs), under the trade name xGnP^®^, grade C, with average diameter between 1 and 2 μm, an average thickness lower than 2 nm, and a specific surface area of about 750 m^2^/g were supplied by XG Sciences Inc (Lansing, MI, USA).

The blends were prepared by melt mixing in a Brabender mixer mod. PLE330 (Brabender, Duisburg, Germany) operating at 270 °C at 60 rpm for 10 min. Before blending, both PET and GnP were dried in a vacuum oven at 120 °C overnight. Table 1 shows the composition of the blends investigated.

The samples used to determine mechanical and rheological properties were prepared by compression molding in a Carver laboratory hydraulic press (Carver, Wabash, IN, USA) at a temperature of 270 °C and a mold pressure of 300 psi for about 3 min. The fibers with diameter between 80 and 400 μm was prepared at 270 °C using the drawing module of a capillary viscometer (Rheologic 1000, CEAST, Turin, Italy).

**Figure 1:**
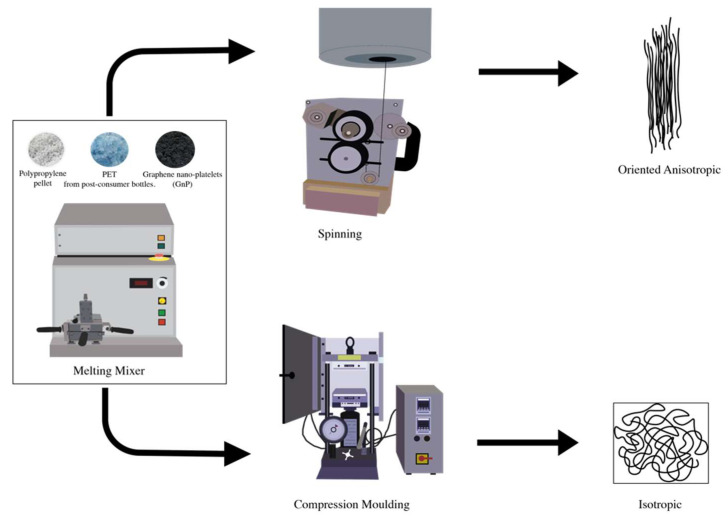
Scheme of the procedure adopted in this work.

Figure 1 depicts the process used in this work.

The rheological characterization in shear flow was performed using a rotational rheometer ARES G2 (TA Instruments, New Castle, DE, USA). The experiments were carried out in parallel plates with a gap of about 1.5 mm and a diameter of 25 mm. The shear viscosity values of the samples were measured at 270 °C from 100 to 0.1 rad/s. The flow curves were also measured in a capillary viscometer (Rheologic 1000, CEAST, Turin, Italy) having a capillary of D = 1 mm and L/D = 40. Due to the large length-to-diameter ratio, Bagley correction was not applied, whereas the Rabinowitsch correction was applied throughout. In order to evaluate the spinnability of these systems, tests in non-isothermal elongational flow were performed in the same instrument with the aid of a drawing module formed by a series of pulleys that grab the hot filament (which gradually cools in the air) and deliver it to an end pulley that rotates at constant speed. The force at break, known as melt strength (MS), of the molten filament was read directly. The breaking stretching ratio (BSR), i.e., the draw ratio at breaking, was calculated as the ratio between the drawing speed at break and the extrusion speed at the die [20].

Mechanical tests were carried out using an Instron (Instron, High Wycombe, U.K.) universal testing machine (mod. 3365) according to ASTM D638 [26]. The crosshead movement speed was of 1 mm/min up to 3 min; subsequently, the crosshead speed was increased to 100 mm/min until specimen failure. Elastic modulus, E; tensile strength, TS; and elongation at break, EB were measured and the data reported were determined as an average of eight samples. Both isotropic sheets and oriented fibers were characterized.

The draw ratio, DR, of the fibers was calculated as:

DR = D_0_^2^/D_F_^2^ (1)

where D_0_ is the diameter of the capillary and D_F_ is the diameter of the fibers.

SEM images were obtained through a Quanta 200F scanning electron microscope (FEI Co., Hillsboro, OR, USA). Before examination by SEM, both sheets and fibers were fractured in liquid nitrogen and gold-sputtered to make them conductive. As for the fibers, the transverse section was observed.

The average dimension of the GnP aggregates was evaluated by measuring length and width (major and minor axes of each particle, considered as an ellipse) of at least twenty particles visible in SEM micrographs, and their area, Ai, was calculated. Then, a probability plot of Ai-s was created for each system. This allowed for determination of the average area, A_eq_, from which the average equivalent diameter, D_eq_, was calculated according to:

D_eq_ = (4A_eq_/π)^1/2^ (2)

## 3. Results and Discussion

**Figure 2:**
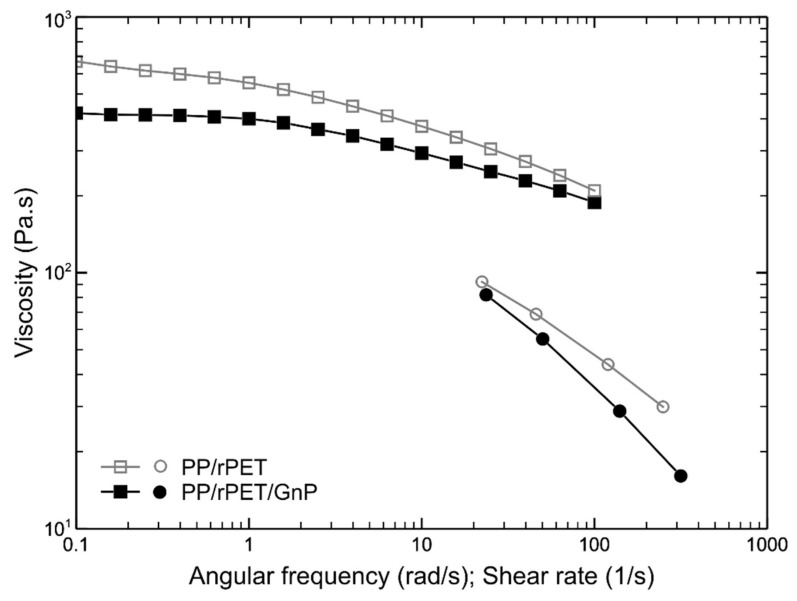
Flow curves of the two systems: data taken from rotational rheometer (square symbols) and capillary viscometer (circle symbols).

In Figure 2, the flow curves of the two polymer systems are reported. The flow curves obtained in the capillary viscometer are not superimposed on the flow curves obtained in the rotational rheometer and do not respect the Cox-Merz rule. This behavior was already reported [20] and is in agreement with results on heterogeneous and multiphase systems [27,28]. Surprisingly, the viscosity of the PP/rPET/GnP blends was lower than that of the PP/rPET blends, and the non-Newtonian behavior of the nanocomposite blend was less pronounced in the test in the rotational viscometer, but much more pronounced in those performed in the capillary viscometer. The lower viscosity of the nanocomposite with respect to the matrix has, however, been observed for other nanocomposite polymer systems [27,29,30], and has been attributed to the presence of the GnP nanoparticles. Moreover, it was observed that GnP nanoparticles act as lubricants. The nanoplatelets, being flexible, deformable, and of nanometric dimensions, can easily exert a lubricating effect on the macromolecular chains of the polymer matrix, reducing their mutual friction and, therefore, the overall viscosity.

The lower viscosity and the lubricating effect of the nanoplatelets can be considered responsible for the less pronounced non-Newtonian effect of the nanocomposite in the test performed in the rotational viscometer, where only a shear flow was involved. The capillary viscometer, on the contrary, involved a convergent flow at the entrance of the capillary. In this flow type, similar to the elongational flow, the macromolecules and the nanoparticles tended to orient along the flow direction. This orientation made the flow of the melt easier. Since this orientation increased with increasing flow rate and was more efficient in the nanocomposite, due to the orientation of the nanoplatelets, this behavior explains the more pronounced non-Newtonian effect observed for the nanocomposite.

**Figure 3:**
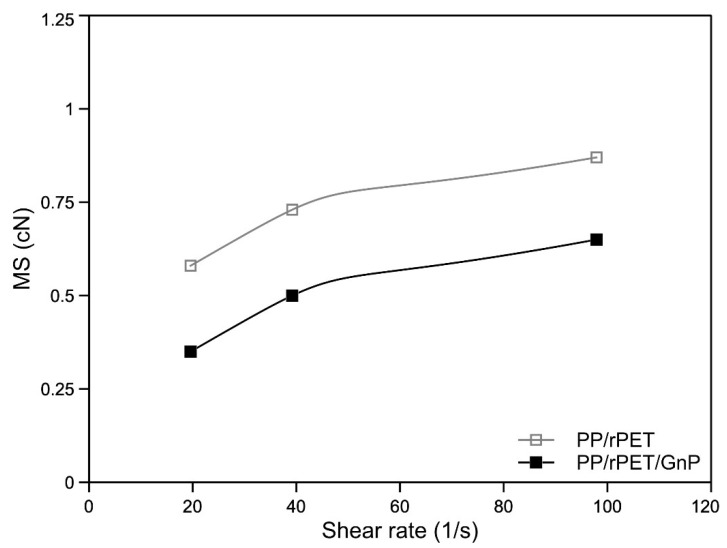
Melt strength (MS) as function of the shear rate of the two systems.

**Figure 4:**
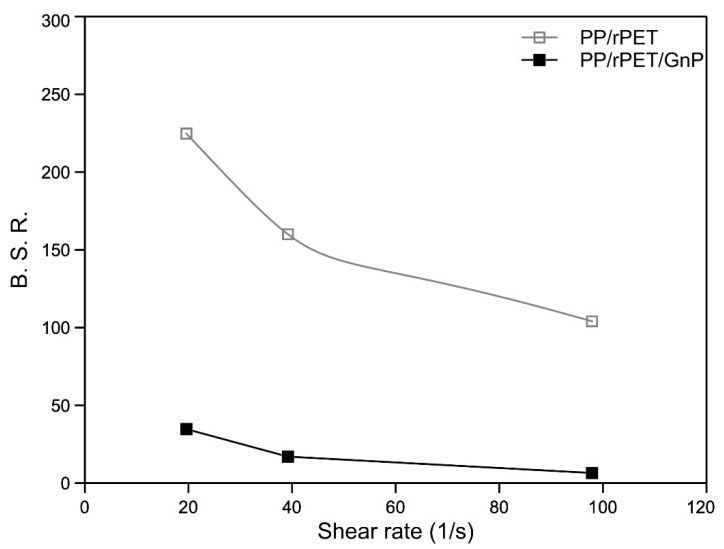
Breaking stretching ratio (BSR) as function of the shear rate of the two systems.

To assess the processability of the formulated systems in processing operations involving a non-isothermal elongational flow, such as film blowing or melt spinning, the evaluation of their rheological behavior when subjected to this kind of flow is of fundamental importance. To this aim, the melt strength (MS) and the breaking stretching ratio (BSR) for both the investigated materials were evaluated, and their variation as a function of the applied shear rate is presented in Figure 3 and Figure 4. MS refers to the force in the molten filament at breaking, while BSR is the ratio between the drawing speed at breaking and the extrusion rate.

The data show that as the shear rate increases, the MS values of both systems increase. In particular, this behavior is consistent with the behavior observed from the flow curve; the presence of GnP causes a decrease in MS. With regard to the BSR, the curves are (as expected) the mirror image of the MS curves: BSR decreases as MS increases.

The values of BSR of the nanocomposite are lower than those of the matrix. This behavior is consistent with the presence of GnP and the heterophasic nature of the sample that forms some defects in the specimen, which can provoke premature ruptures of the melt.

**Figure 5:**
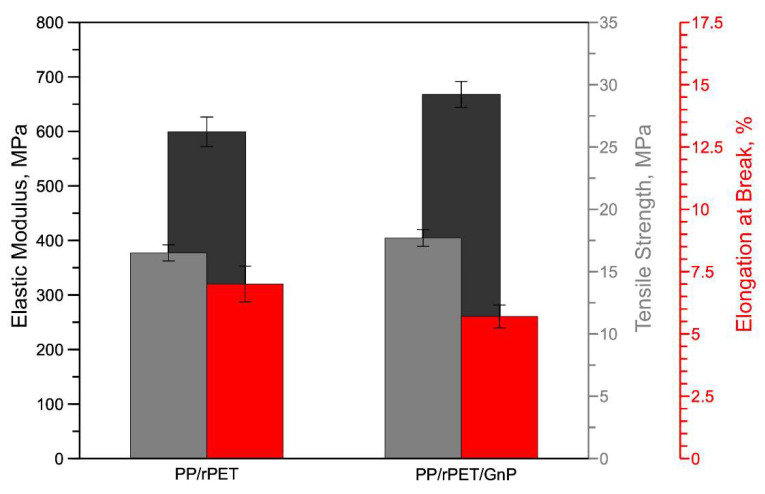
Tensile properties of isotropic sheets.

In Figure 5, the tensile properties of the isotropic, compression-molded sheets are reported for the two samples.

The histogram shows that the introduction of GnP causes a slight increase in the elastic modulus. More specifically, the elastic modulus of the PP/rPET blend increases from 599 ± 27 to 668 ± 24 MPa for PP/rPET/GnP. This result highlights that GnPs exhibit a slight reinforcing action in the PP/rPET/GnP blend compared to the PP/rPET blend. Similarly, the tensile strength shows a slight increase of about 7% compared to the PP/rPET blend. On the contrary, the elongation at break remains almost unchanged. More precisely, the elongation at break of the PP/rPET blend shows average values of 7 ± 0.7%, while the PP/rPET/GnP blend shows average values of about 6 ± 0.5%. The slight effect of the GnP on the elongation at break could be, attributed to the lubricating effect of the graphene nanoplatelets.

**Figure 6:**
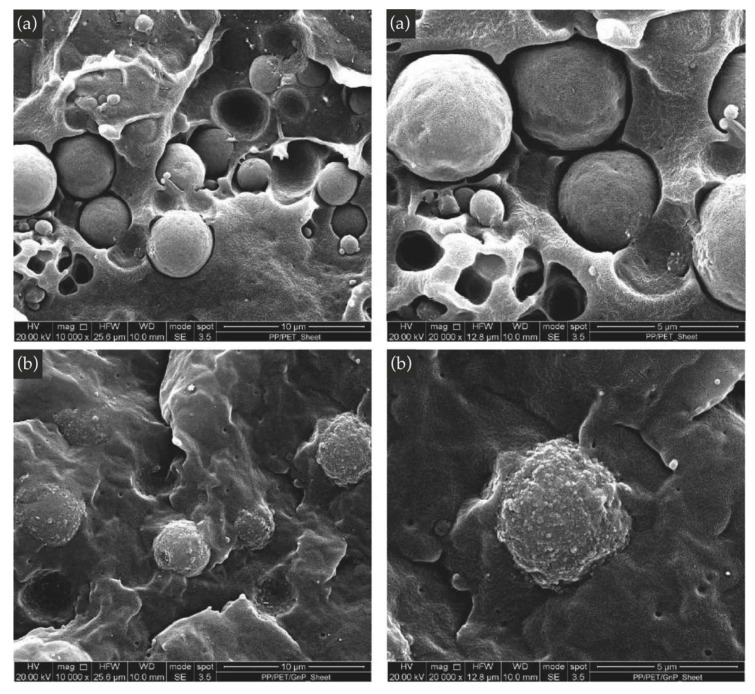
SEM micrograph of the blend (**a**) and of the nanocomposite (**b**) at different magnifications.

In Figure 6a,b, the SEM micrographs of the sheets of the two systems are shown. From the images of the blend, two distinct incompatible phases are observed, with the PET phase easily identifiable. The PET particles are spherical and well-separated by the PP matrix confirming the poor morphology of this biphasic blend with poor adhesion between the two phases. The diameters of the spherical rPET particles are in the range of 1–4 μm.

The picture is different in the PP/rPET/GnP nanocomposite blend. The dimensions of the PET particles are similar to those observed in the blend, but the GnPs seem to be mainly distributed around the droplets of the PET phase giving rise to a sort of honey-like appearance. Furthermore, GnPs are almost not visible in the PP phase suggesting that the particles are mainly located in the PET phase. However, it is important to note that the compounding process was able to break up the GnP aggregates present in the neat powder [31], obtaining GnP particles having submicrometric dimensions, even if located only in the PET phase. In particular, the average equivalent diameter of the GnP aggregates (evaluated, as reported in the experimental section, by measuring the dimensions of at least twenty particles) is in the range of about 150–300 nm (Figure S1 in the Supplementary Materials). Therefore, GnP particles with submicrometric dimensions are well-dispersed locally in the PET phase. Overall, the composite system shows the filler localized in large aggregates with dimensions in the range of 1–4 μm, i.e., the diameters of the spherical rPET particles. This could explain the low reinforcing effect of the GnP as revealed by the slight increase in the elastic modulus of isotropic nanocomposite samples.

Moreover, these observations could explain the lubricating effect exerted by the GnPs. The presence of GnP on the surface of PET particles could decrease the friction between the two phases. Therefore, this peculiar morphology could be responsible for the almost lack of variation in the elongation at break, although there was poor adhesion between the two polymeric phases.

**Figure 7:**
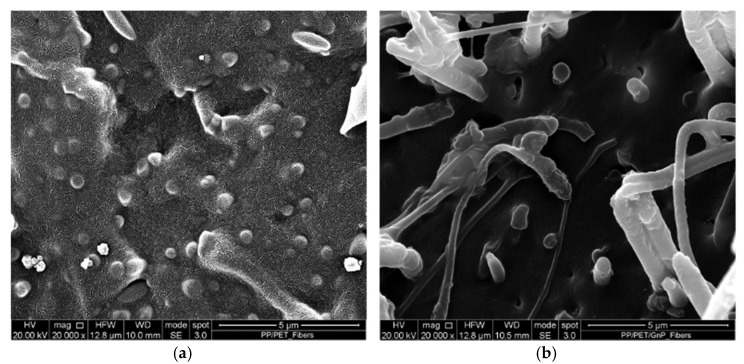
SEM micrograph of fibers of the blend (**a**) and the nanocomposite (**b**).

Figure 7 shows the SEM micrographs of the fibers with DR = 90 of the two polymers systems. As reported in the experimental section, the micrographs show transverse sections of the fibers.

The morphology of both anisotropic oriented fibers dramatically changes with respect to the isotropic sheets in Figure 6. The first comment is that the two phases seem more adherent than in the isotropic sheet. Some compatibilization driven by the orientation was already shown in a previous paper [28] and was attributed to the processing method, in particular to the orientation achieved by the elongational flow, which can lead to improvements normally achieved by adding a compatibilizer.

The PET particles are elongated, and the microfibrils in these fibers (with a draw ratio of 90) present a diameter of about 200–600 nm with a high length/diameter ratio. In the blend, these PET microfibrils are embedded in the PP matrix, while the same microfibrils are elongated and pulled out of the matrix in the case of the nanocomposite system. The elongation of the PET particles is the result of the non-isothermal elongation flow during the spinning. The pull-out of the same fibers in the nanocomposite sample could be attributed to the lubricating effect of the GnPs on the skin of the PET particles that allows the PET microfibrils to be pulled out of the PP matrix. The dimension of the GnP aggregates is about 150–250 nm and slightly smaller than those observed in the isotropic sample. This reduction in size is certainly due to the action of the elongational flow, which is able to disintegrate the nanoparticles giving rise to smaller aggregates [9].

**Figure 8:**
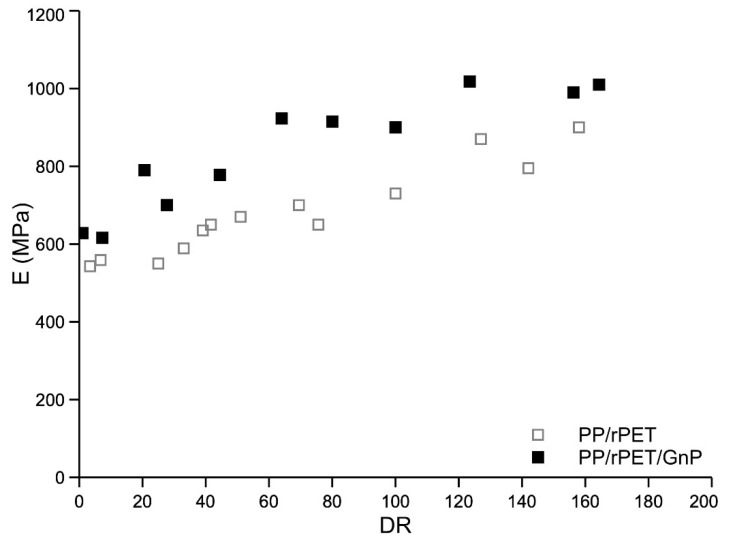
Elastic modulus as a function of the draw ratio.

**Figure 9:**
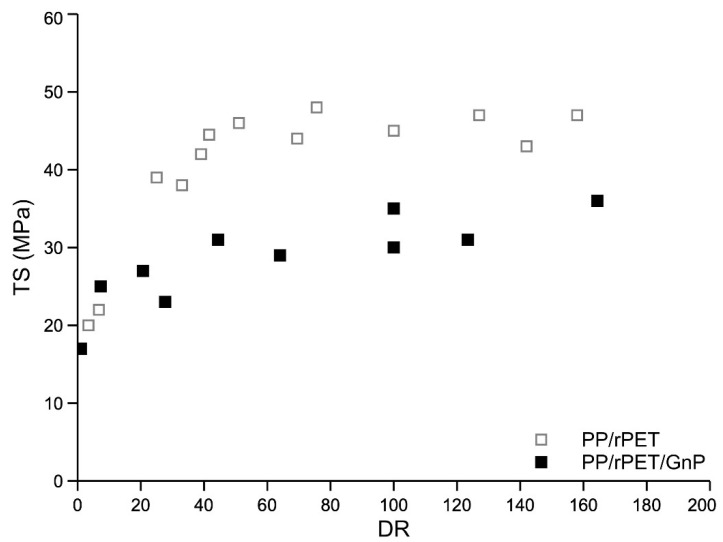
Tensile strength as a function of the draw ratio.

**Figure 10:**
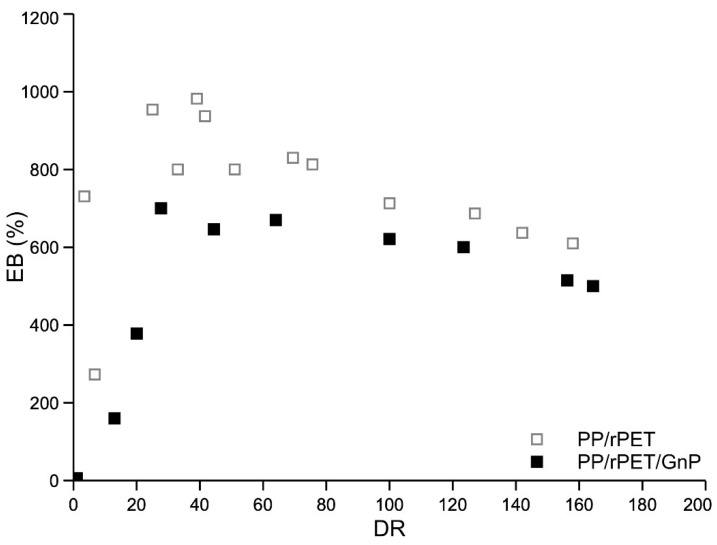
Elongation at break as a function of the draw ratio.

In Figure 8, Figure 9 and Figure 10, the curves of elastic modulus (E), tensile strength (TS), and elongation at break (EB) of the fibers of the two systems, as a function of the draw ratio, are reported, respectively.

Elastic modulus and tensile strength increase with the draw ratio, while the elongation at break increases at low draw ratio values and then decreases with increasing the draw ratio. The complex trend of the elongation curves at break has been already found and discussed for other similar multiphase systems [10,32,33]. Compared with isotropic samples, the dramatic increase of the elongation at break of anisotropic-oriented nanocomposite fibers was interpreted considering that the dispersed PET particles, oriented along the spinning direction, can facilitate the deformation of the macromolecules, mitigating the stress along the sample and avoiding a premature break. Both systems are fragile in the isotropic state but become ductile because of the orientation achieved during the spinning. A fragile-ductile transition driven by the orientation is observed for both systems. The elastic modulus of the nanocomposites is higher than that of the blend due to the reinforcing effect of the inert nanoparticles. On the contrary, both tensile strength and of the nanocomposite blend are lower than those of the unreinforced blend. The reduction in the elongation at break is a typical effect of the addition of inert particles in a polymer matrix. This reduction in the elongation at break is, in turn, responsible for the lower value of the tensile strength. Indeed, The lower value of the elongation at break results in a premature break of the sample and, then, to a lower value of the tensile strength.

## 4. Conclusions

In this work, PP/rPET and PP/rPET/GnP nanocomposite blends were prepared by melt mixing, and then PP/rPET and PP/rPET/GnP isotropic sheets were prepared by compression molding. The anisotropic fibers were spun by using a drawing module of a capillary viscometer. Morphological, mechanical, and rheological properties were examined.

It was observed that the presence of GnPs in a PP/rPET blend significantly modifies the rheological and mechanical behavior of the blend. The shear viscosity and the non-Newtonian behavior of the nanocomposite are reduced by the presence of the nanoparticles. This behavior was correlated with the lubricant effect of the GnPs that is preferably located around the dispersed PET particles. The same effect was observed in elongational flow where the melt strength is also lower in the nanocomposite. The presence of the GnPs slightly improves the mechanical properties of the isotropic sheets of the blend. The elastic modulus and tensile strength of the fibers strongly increase with the draw ratio for both systems, due to the orientating effect of the elongational flow. While the elastic modulus of the nanocomposite is larger with respect to that of the blend, both tensile strength and elongation at break remain higher for the unreinforced blend.

Although both systems are fragile in the isotropic state, the elongation at break increases with the draw ratio and both systems become ductile. A fragile–ductile transition driven by the orientation is then observed for both systems.

## Figures and Tables

**Table 1:** Composition of the studied blends.

Blends	PP (wt %)	rPET (wt %)	GnP (wt %)
PP/rPET	75	25	-
PP/rPET/GnP	75	25	2

## Data Availability

The data presented in this study are available on request from the corresponding author.

## Supplementary Materials

The following are available online at https://www.mdpi.com/article/10.3390/nano11113058/s1, Figure S1: SEM images of the aggregates of GnPs measured at 40,000× magnification: (**a**) isotropic sheets and (**b**) anisotropic fibers.
