# Supplementary material for: Morphology, Rheological and Mechanical Properties of Isotropic and Anisotropic PP/rPET/GnP Nanocomposite Samples
Source: Nanomaterials (Basel). 2021 Nov 13;11(11):3058. doi: 10.3390/nano11113058 (PMC8625529; doi:10.3390/nano11113058)
Supplement: Supplementary file 1 [file nanomaterials-11-03058-s001.zip › nanomaterials-1427784-supplementary.pdf]

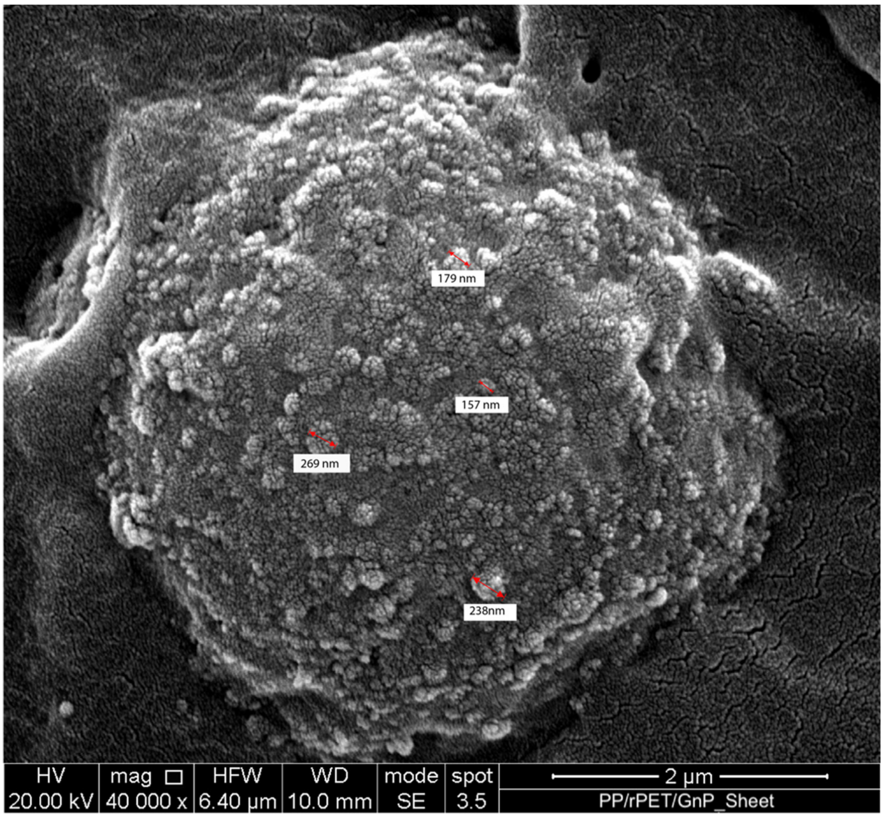

(**a**)

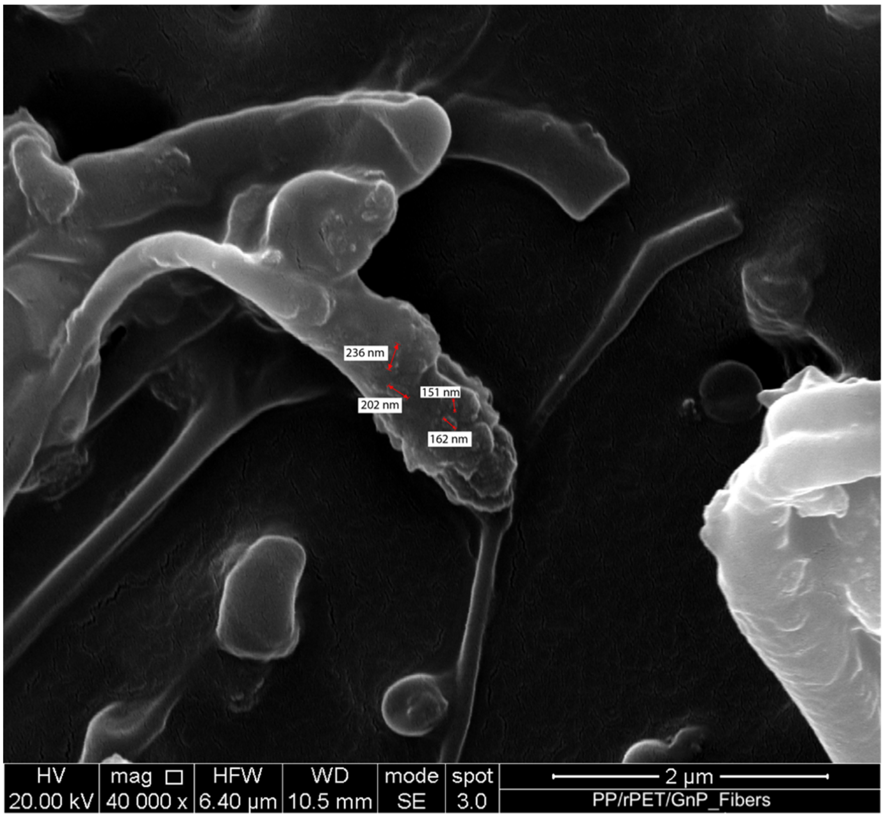

(**b**)

Figure S1. SEM imagine of the aggregates of GnP measured at 40,000× magnification: (**a**) isotropic sheets, (**b**) anisotropic fibers.
